# *Alloscopus ramanai* sp. nov. (Orchesellidae, Heteromurinae), a new Collembola species from central Thailand, with complete mitochondrial genome and phylogenetic placement

**DOI:** 10.3897/BDJ.14.e173157

**Published:** 2026-01-23

**Authors:** Areeruk Nilsai, Sopark Jantarit, Tadsanai Jeenthong, Matsapume Detcharoen, Weeyawat Jaitrong

**Affiliations:** 1 Division of Biological Science, Faculty of Science and Digital Innovation, Thaksin University, Pa Phayom, Phatthalung, Thailand Division of Biological Science, Faculty of Science and Digital Innovation, Thaksin University, Pa Phayom Phatthalung Thailand; 2 Excellence Center for Biodiversity of Peninsular Thailand, Faculty of Science, Prince of Songkla University, Hat Yai, Songkhla, Thailand Excellence Center for Biodiversity of Peninsular Thailand, Faculty of Science, Prince of Songkla University, Hat Yai Songkhla Thailand; 3 Office of Natural Science Research, National Science Museum Thailand, 39, Moo 3, Khlong 5, Khlong Luang, Pathum Thani, Thailand Office of Natural Science Research, National Science Museum Thailand, 39, Moo 3, Khlong 5, Khlong Luang Pathum Thani Thailand; 4 Division of Biological Science, Faculty of Science, Prince of Songkla University, Hat Yai, Songkhla, Thailand Division of Biological Science, Faculty of Science, Prince of Songkla University Hat Yai, Songkhla Thailand

**Keywords:** Entomobryoidea, mitogenome, new species, taxonomy, phylogeny

## Abstract

**Background:**

*Alloscopus* is one of the genera within the subfamily Heteromurinae, recently recorded in Thailand and is currently represented by six species from two regions of the country. In the northern part, *A. tetracanthus* Börner, 1906 and *A. thailandensis* Mari Mutt, 1985 have been recorded from forested habitats. In the southern part, *A. whitteni* Jantarit & Sangsiri, 2020, *A. namtip* Jantarit & Sangsiri, 2020 and *A. jantapasoae* Jantarit, Nilsai & Manee, 2025 have been reported from a cave habitat, while *A. sago* Jantarit & Manee, 2025 was recently described from a sago palm forest.

**New information:**

A new species, *Alloscopus ramanai* **sp. nov.**, is described from central Thailand, based on an integrative taxonomic approach combining morphological and molecular data. The new species closely resembles *A. tetracanthus* Börner, 1906, sharing several diagnostic characters including a dark red ocular patch and PAO shape and the number of M and S series chaetae on the dorsal head. Additional similarities include the number of spiniform labral papillae, labial basis chaetae, the number of pseudopores on the manubrium, the number of central and lateral macrochaetae on Th.II, the number of central macrochaetae on Th.III and Abd.IV. However, *A. ramanai* **sp. nov.** can be clearly distinguished from *A. tetracanthus* by a unique combination of traits, including the number of lateral macrochaetae on Abd. III and Abd. IV and the number of chaetae on the anterior side of the ventral tube. A detailed diagnosis and illustrations of the new species are provided herein. A key for species of *Alloscopus* in Thailand is also included. The complete mitochondrial genome of *A. ramanai* **sp. nov.** is 14,757 bp in length and comprises 13 concatenated protein-coding genes (PCGs), 22 transfer RNA (tRNA) genes and two ribosomal RNA (rRNA) genes. Phylogenetic analysis, based on mitochondrial genome data, indicates that *A. ramanai* **sp. nov.** forms a sister lineage to *Alloscopus bannaensis* Zhang, 2020. The description of this new species contributes to a more comprehensive understanding of Heteromurinae diversity in Thailand and underscores the need for expanded mitogenomic sampling across Collembola.

## Introduction

The family Orchesellidae *sensu* Zhang et al. (2019) comprises four subfamilies: Nothobryinae *sensu* Nunes et al. (2020), Orchesellinae *sensu* Zhang and Deharveng (2015), Heteromurinae *sensu* Zhang and Deharveng (2015) and Bessoniellinae *sensu* Zhang and Deharveng (2015) with approximately 280 species in 19 genera (Bellinger et al. 1996-2025, Godeiro et al. 2023b). Amongst these, Heteromurinae is the largest subfamily distinguished by five antennal segments (Ant. I subdivided into Ia and Ib) or six segments (Ant II also divided into IIa and IIb), a dorsal abdominal segment IV/III length ratio of less than two times, body scales present and tergal sens formula as 2,2/1,3,3 (Cipola et al. (2016), Zhang et al. (2020), Godeiro et al. (2023b). Initially classified within Orchesellinae (Zhang et al. 2019), Heteromurinae was recognised as a distinct subfamily by Zhang and Deharveng (2015) following a revision based on sensillar patterns. This subfamily is further divided into two tribes, Heteromurini and Mastigocerini, which are differentiated by the types of scales present on the body and appendages (Zhang and Deharveng 2015, Cipola et al. 2016). The classification of Heteromurini was revised by Zhang et al. (2020), based on phylogenetic relationships and the transformation patterns of S-chaetae on abdominal segment V. Following the inclusion of additional genera recognised by Handschin (1924), Mandal (2018) and Bellini et al. (2021), the tribe currently comprises eight genera: *Alloscopus Börner 1906, Dicranocentrus* Schött 1893, *Falcomurus Mandal 2018, Heteromurus Wankel 1860*, *Heteromurtrella Mari-Mutt 1979*, *Mastigoceras Handschin 1924*, *Pseudodicranocentrus Mari-Mutt 1981* and *Sinodicranocentrus* Zhang et al. 2020. *Alloscopus* is a rather small genus within the subfamily Heteromurinae, comprising 17 nominal species worldwide (Bellinger et al. 1996-2025, Cipola et al. 2016, Jantarit et al. 2025). It was originally established as a subgenus of *Heteromurus* by Börner (1906) and later advanced to genus rank. Members of *Alloscopus* are distributed across the Pantropical Region and inhabit a variety of moist environments, including forest soil, caves, humus and mosses and even canopy microhabitats (Cipola et al. 2016, Jantarit et al. 2016, Alviola et al. 2024, Jantarit et al. 2025). This genus is characterised by five-segmented antennae (with the subdivision of the first antennal segment and the annulation of antennal segments III and IV), the presence of a post-antennal organ (PAO) on the dorsal side of the head and the presence of dental spines. The dorsal head chaetotaxy typically lacks the S2 macrochaetae, while the post-occipital series may either include or lack the Pa5 macrochaeta. Regarding body chaetotaxy, all species of *Alloscopus* generally exhibit a stable pattern, with abdominal segment I (Abd. I) bearing 3+3 macrochaetae and abdominal segment II (Abd. II) bearing 1+1 central macrochaetae (Cipola et al. 2016, Jantarit et al. 2016, Jantarit and Sangsiri 2020, Jantarit et al. 2025).

In the present study, we describe a new species of *Alloscopus* from Pathum Thani Province, central Thailand. We also present the complete mitogenome of the new species, *Alloscopus ramanai* **sp. nov.** and conduct a phylogenetic analysis to clarify its evolutionary relationships with other congeners. These findings contribute to a more comprehensive understanding and validation of the phylogenetic placement of the subfamily Heteromurinae within the family Orchesellidae.

## Materials and methods

**Specimen collection and preparation**

**Figure 1.**
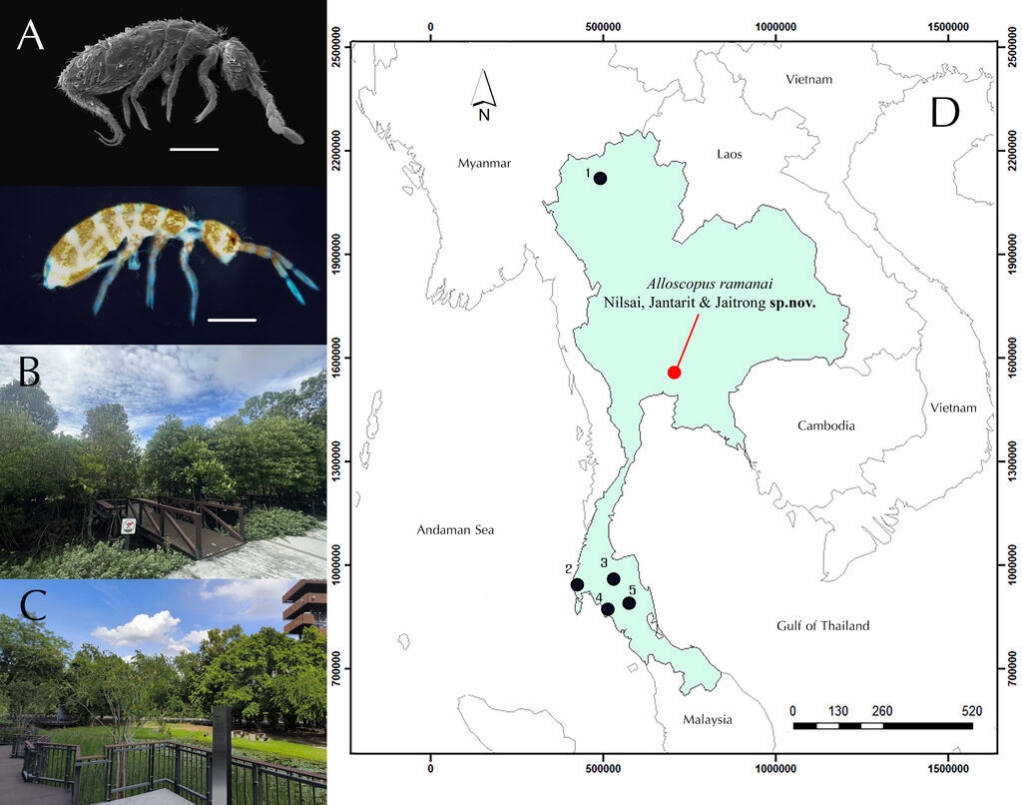
**A** Habitus of *Alloscopus ramanai* **sp. nov.** (SEM image, upper; wet specimen, lower). Scale bars = 0.5 mm; **B–C** The type locality of the new species, a forest plantation of the Natural History Museum of the National Science Museum Thailand (THNHM); **D** The localities of *Alloscopus* recorded in Thailand. 1, *A. tetracanthus* Börner, 1906 and *A. thailandensis* Mari Mutt, 1985. 2, *A. whitteni* Jantarit & Sangsiri, 2020. 3, *A. namtip* Jantarit & Sangsiri, 2020. 4, *A. jantapasoae* Jantarit, Nilsai & Manee, 2025. 5, *A. sago* Jantarit & Manee, 2025.

The specimens were collected by visual searching using an aspirator and preserved in 95% ethanol. All samples were obtained in the debris and leaf litter near a small pond and a swamp area within the forest plantation of the National Science Museum Thailand (THNSM), Khlong Luang District, Pathum Thani Province, central Thailand (Fig. 1D). Springtails were euthanised following the ethical protocols of Thaksin University (COA No. TSU 2025-006).

For taxonomic examination, specimens were cleared in Nesbitt’s fluid and mounted in Hoyer’s medium for observation under a light microscope. External morphological features were examined using a Leica DM1000 LED microscope, equipped with phase-contrast. Selected body structures were further examined and imaged using a scanning electron microscope (SEM; FEI Quanta 450 FEG), equipped with an Oxford Instruments X-Max 50 energy-dispersive spectroscopy (EDS) system. The photograph of a wet specimen was taken with a Canon 5D digital camera fitted with a Canon MP-E 65 mm Macro Photo Lens and Canon Extender EF 2.0× III (Canon, Tokyo, Japan) and a Stack-Shot Macrorail (Cognisys Inc, MI, USA).

For the dorsal body chaetotaxy, we followed the terminology of Szeptycki (1979) and Zhang et al. (2019). Head chaetotaxy was described using the notation of Mari-Mutt (1977) and Soto-Adames (2008). All chaetotaxy details are depicted on a single side of the body, from the head to abdominal segment IV (Abd. IV). The description of the labial palp followed Fjellberg (1999) and the labial chaetae were depicted according to Gisin (1967) and Cipola et al. (2016).

The symbols used for chaetotaxy in the figures are as follows: large circle = macrochaeta; medium circle = mesochaeta; small circle = microchaeta; cross (X) = trichobothrium; circle with a slash (Ø) = pseudopore.

The abbreviations used for chaetotaxy in description are as follows: Abd. — abdominal segment(s); Ant. — antennal segment (s); mac — macrochaeta(e); mes —mesochaeta(e); mic—microchaeta(e); ms — S-microchaeta(e); sens — ordinary S-chaeta(e); Th. — thoracic segment(s); tric — trichobotrium; tita — tibiotarsus and psp — pseudopore. Antennal segments I and II subdivisions are: “a” to the proximal subarticle and “b” to the distal one and SEM— Scanning Electron Microscope.

The holotype and some paratypes are deposited in the Animal and Plant Herbarium, Faculty of Science and Digital Innovation, Thaksin University, Thailand (APH-TSU) and four paratypes are in the Natural History Museum of the National Science Museum Thailand (THNHM) and two paratypes are at the Princess Maha Chakri Sirindhorn Natural History Museum (NHM-PSU), Prince of Songkla University (HatYai, Songkhla, Thailand).

**DNA extraction, Sequencing, Mitogenome assembly and Mitochondrial genome analyses**

Four individuals were selected for non-destructive DNA extraction. Genomic DNA was extracted separately from each specimen using the QIAGEN DNeasy Blood and Tissue Kit (QIAGEN, Hilden, Germany), following the manufacturer’s protocol. DNA quality was assessed using 1% agarose gel electrophoresis and DNA concentration was measured using a spectrophotometer. As the DNA yield per individual was low, equimolar amounts of DNA from the four extractions were pooled to generate a single library for sequencing.

For Oxford Nanopore sequencing, DNA was end-prepped for ligation using the NEBNext Companion Module for Oxford Nanopore Technologies Ligation Sequencing (NEB, MA, USA), as per the manufacturer’s instructions. The DNA was subsequently cleaned with AMPure XP Beads (Beckman Coulter, IN, USA) before being ligated to adaptors using the SQK-LSK114 ligation sequencing kit (Oxford Nanopore Technologies, Oxford, UK). The final sequencing library quantity was measured with a Qubit 4 fluorometer with dsDNA high sensitivity kit (Thermo Fisher Scientific, MA, USA). The library was loaded onto the R10.4.1 flow cell and sequenced for 24 hours on a MinION device.

Base calling was performed on the reads using Dorado v.0.7.3 (Oxford Nanopore Technologies, Oxford, UK) with a super-accurate model (dna_r10.4.1_e8.2_400bps_sup@v5.0.0). Fastp v.0.20.1 was then used to remove low-quality reads with default parameters (Chen 2023). We utilised Diamond v.2.1.10.164 to extract putative mitochondrial reads by aligning them to the protein sequences of *Homidia koreana* (GenBank accession MZ934725.1), *Dicranocentrus wangi* (NC_046887.1) and *Seira pallidipes* (OR115504.1). The mitochondrial reads were *de novo* assembled using Flye v.2.9.5-b1801 (Kolmogorov et al. 2019) and the resulting assembly was inspected with Bandage v.0.8.1 (Wick et al. 2015). The assembly was subsequently polished twice using pilon v.1.24 (Walker et al. 2014). Finally, the mitochondrial genome was annotated using Mitos2 v.2.1.9 (Bernt et al. 2013). Data availability is submitted with submission ID: 2999622. Sequencing raw reads was deposited on NCBI SRA under project PRJNA1313599.

For the mitogenome-based phylogenetic analysis, the 13 mitochondrial protein-coding genes from *A. ramanai* **sp. nov.** and seven publicly available Entomobryomorpha mitogenomes, *Orchesella villosa* (EU016195.1), *Orchesella cincta* (KT985987.1), *Heteromurus nitidus* (MT611220.1), *Troglopedetes dispersus* (MT914176.1), *Cyphoderus albinus* (NC046888.1), *Alloscopus bannaensis* (OK037063.1) and *Orchesella flavescens* (OZ035959.1), were included. Each protein-coding gene (PCG) was aligned separately using Clustal Omega v.1.1.0 (Sievers et al. 2011) and alignments were concatenated for Maximum-Likelihood (ML) inference in IQ-TREE v.2.3.6 (Minh et al. 2020) with 10,000 ultrafast bootstrap replicates. The dataset was partitioned by gene and ModelFinder (Kalyaanamoorthy et al. 2017), as implemented in IQ-TREE, was used to select the best-fit substitution model for each partition under the Bayesian Information Criterion (BIC) using an edge-linked proportional partition model (linked branch lengths across partitions, with separate substitution models and separate rates across sites). The selected models were HKY+F+G4 for atp6, TVM+F+I+G4 for atp8 + nad2 + nad6, GTR+F+R3 for cox1 + cox2 + cox3 + cob, K3Pu+F+I+G4 for nad1 + nad4 + nad4l + nad5 and HKY+F+I+G4 for nad3. Codon-position partitioning was not applied in this mitogenome-only analysis. *Cyphoderus albinus* and *T. dispersus* were used as outgroups.

We also conducted a phylogenetic analysis of *A. ramanai* **sp. nov.** and other Entomobryomorpha species using the nuclear 28S rRNA marker and the mitochondrial COI and 16S rRNA markers. Oxford Nanopore reads were mapped to the corresponding marker reference sequences downloaded from NCBI (Suppl. material 2) using minimap2 v.2.28-r1209 with the map-ont preset. The mapped reads were then assembled with Flye v.2.9.6-b1802 using default parameters. Assemblies were validated by nucleotide BLAST searches against the core_nt database to confirm marker identity. Sequences were aligned with ClustalW as implemented in MEGA v.12.0.11 and the resulting alignments were concatenated into a combined dataset of 1,822 columns. Maximum-Likelihood inference was performed in IQ-TREE v.3.0.1 with 1,000 ultrafast bootstrap replicates, using the GTR+F+I+G4 model for COI, TVM+F+I+G4 for 28S and TVM+F+G4 for 16S rRNA. *Ceratophysella denticulata* and *Hypogastrura reticulata* (Poduromorpha) were used as outgroups. The final tree was visualised in FigTree v.1.4.4.

## Taxon treatments

### Alloscopus ramanai

Nilsai, Jantarit & Jaitrong sp. nov.

B366DA4D-80AB-57A1-983D-9D385E808AB8

D8BD8642-7729-4A4D-AEB4-15062CA1A455

PRJNA1313599

#### Materials

- **Type status:** Holotype. **Occurrence:** recordNumber: # THA_AN_PKN01_1-001; recordedBy: Nilsai, A, Jantarit, S & Jaitrong, W; individualCount: 1; sex: Female; lifeStage: adult; occurrenceID: 715D8AE6-3B8E-5488-8EBB-49E70D7B6B75; **Taxon:** scientificName: Collembola; phylum: Arthropoda; class: Collembola; family: Orchesellidae; genus: Alloscopus; **Location:** continent: Asia; country: Thailand; countryCode: TH; county: Pathum Thani; locality: Khlong Luang; verbatimLocality: A forest plantation located in a protected zone of the National History Museum Thailand (THNSM) at the Ramanai 9 building in Pathum Thani Province; verbatimElevation: 4-5; minimumElevationInMeters: 3; maximumElevationInMeters: 6; verbatimCoordinates: 14°3'39.657"N, 100°43'1.782"E; verbatimCoordinateSystem: degrees minutes seconds; verbatimSRS: WGS84; coordinateUncertaintyInMeters: 30; **Identification:** identifiedBy: Nilsai, A, Jantarit, J & Jaitrong, W; **Event:** samplingProtocol: Aspirator (Nilsai, A & Jeenthong, T); year: 2025; month: iv; day: 13; habitat: the leaf litter and soil; **Record Level:** language: en; **Material Entity:** disposition: In the collection of the Animal and Plant Herbarium, Faculty of Science and Digital Innovation, Thaksin University, Thailand (APH-TSU)
- **Type status:** Paratype. **Occurrence:** recordNumber: THNHM-I-0003518 to THNHM-I-00030521; recordedBy: Nilsai, A, Jantarit, S & Jaitrong, W; individualCount: 4; sex: 2 females; lifeStage: 2 adults, 2 subadults; occurrenceID: E6293B1F-E434-58BB-A04B-26BBACB8AA54; **Taxon:** phylum: Arthropoda; class: Collembola; family: Orchesellidae; genus: Alloscopus; **Location:** continent: Asia; country: Thailand; countryCode: TH; county: Pathum Thani; locality: Khlong Luang; verbatimLocality: A forest plantation located in a protected zone of the National History Museum Thailand (THNSM) at the Ramanai 9 building in Pathum Thani Province; verbatimElevation: 4-5; minimumElevationInMeters: 3; maximumElevationInMeters: 6; verbatimCoordinates: 14°3'39.657"N, 100°43'1.782"E; verbatimCoordinateSystem: degrees minutes seconds; verbatimSRS: WGS84; coordinateUncertaintyInMeters: 30; **Identification:** identifiedBy: Nilsai, A, Jantarit, J & Jaitrong, W; **Event:** samplingProtocol: Aspirator (Nilsai, A & Jeenthong, T); year: 2025; month: iv; day: 13; habitat: the leaf litter and soil; **Record Level:** language: en; **Material Entity:** disposition: In the collection of Natural History Museum, Thailand (THNHM)
- **Type status:** Paratype. **Occurrence:** recordNumber: # THA_AN_PKN01-1-002 and # THA_AN_PKN01-1-003; recordedBy: Nilsai, A, Jantarit, S & Jaitrong, W; individualCount: 2; sex: 1 female, 1 subadult; lifeStage: 1 adults, 1 subadults; occurrenceID: ECB9DAEE-BEB9-5D9C-BEA0-E86D98564CFD; **Taxon:** phylum: Arthropoda; class: Collembola; family: Orchesellidae; genus: Alloscopus; **Location:** continent: Asia; country: Thailand; countryCode: TH; county: Pathum Thani; locality: Khlong Luang; verbatimLocality: A forest plantation located in a protected zone of the National History Museum Thailand (THNSM) at the Ramanai 9 building in Pathum Thani Province; verbatimElevation: 4-5; minimumElevationInMeters: 3; maximumElevationInMeters: 6; verbatimCoordinates: 14°3'39.657"N, 100°43'1.782"E; verbatimCoordinateSystem: degrees minutes seconds; verbatimSRS: WGS84; coordinateUncertaintyInMeters: 30; **Identification:** identifiedBy: Nilsai, A, Jantarit, J & Jaitrong, W; **Event:** samplingProtocol: Aspirator (Nilsai, A & Jeenthong, T); year: 2025; month: iv; day: 13; habitat: the leaf litter and soil; **Record Level:** language: en; **Material Entity:** disposition: In the collection of the Animal and Plant Herbarium, Faculty of Science and Digital Innovation, Thaksin University, Thailand (APH-TSU)
- **Type status:** Paratype. **Occurrence:** recordedBy: Nilsai, A, Jantarit, S & Jaitrong, W; individualCount: 2; lifeStage: 2 subadults; occurrenceID: 132709A5-3D36-59FD-99E2-057B20A769F9; **Taxon:** phylum: Arthropoda; class: Collembola; family: Orchesellidae; genus: Alloscopus; **Location:** continent: Asia; country: Thailand; countryCode: TH; county: Pathum Thani; locality: Khlong Luang; verbatimLocality: A forest plantation located in a protected zone of the National History Museum Thailand (THNSM) at the Ramanai 9 building in Pathum Thani Province; verbatimElevation: 4-5; minimumElevationInMeters: 3; maximumElevationInMeters: 6; verbatimCoordinates: 14°3'39.657"N, 100°43'1.782"E; verbatimCoordinateSystem: degrees minutes seconds; verbatimSRS: WGS84; coordinateUncertaintyInMeters: 30; **Identification:** identifiedBy: Nilsai, A, Jantarit, J & Jaitrong, W; **Event:** samplingProtocol: Aspirator (Nilsai, A & Jeenthong, T); year: 2025; month: iv; day: 13; habitat: the leaf litter and soil; **Record Level:** language: en; **Material Entity:** disposition: In the collection of the Princess Maha Chakri Sirindhorn Natural History Museum (NHM-PSU)

#### Description

**Figure 2.**
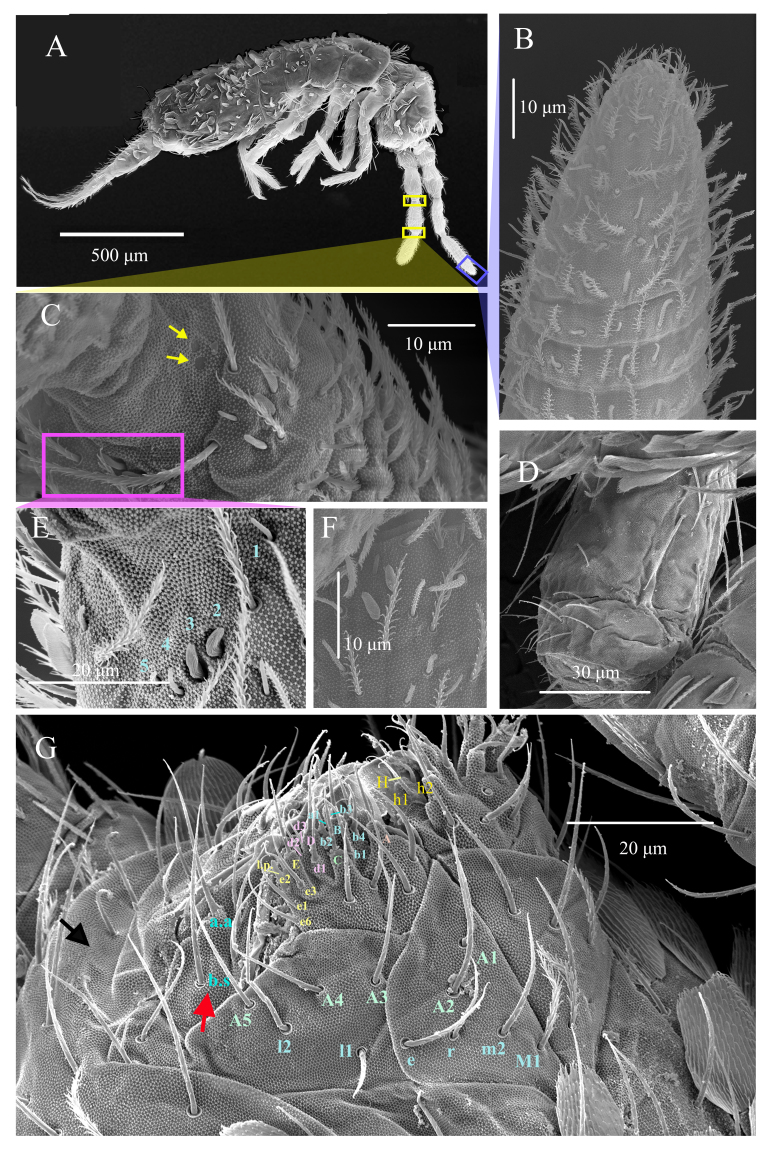
*Alloscopus ramanai* **sp. nov.** (SEM) **A** Habitus; **B** Tip of Ant. IV; **C** Position of pseudopores on Ant. III; **D** Anterior-lateral view of ventral tube; **E** Right Ant. III organ; **F** Right Ant. II organ; **G** Ventral part of the head shows labial basis, maxillary outer lobe (red arrow) and labial papillae (black arrow).

**Figure 3.**
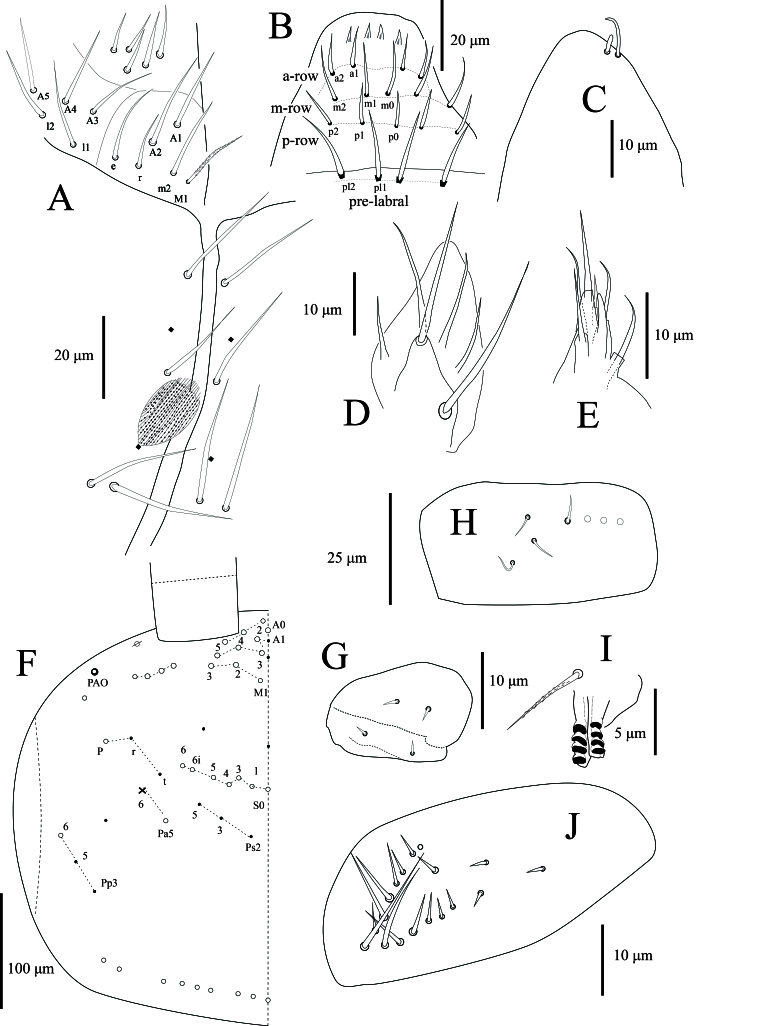
*Alloscopus ramanai* **sp. nov. A** Head ventral chaetotaxy anteriorly; **B** Labial papillae; **C** Tip of Ant. IV with subapical organite and guard chaeta; **D** Maxillary outer lobe (left side); **E** Labial palp (right side); **F** Dorsal cephalic chaetotaxy (left side); **G** Genital plate of female; **H** Left Ant Ia; **I** Tenaculum; **J** Trochanteral organ.

**Figure 4.**
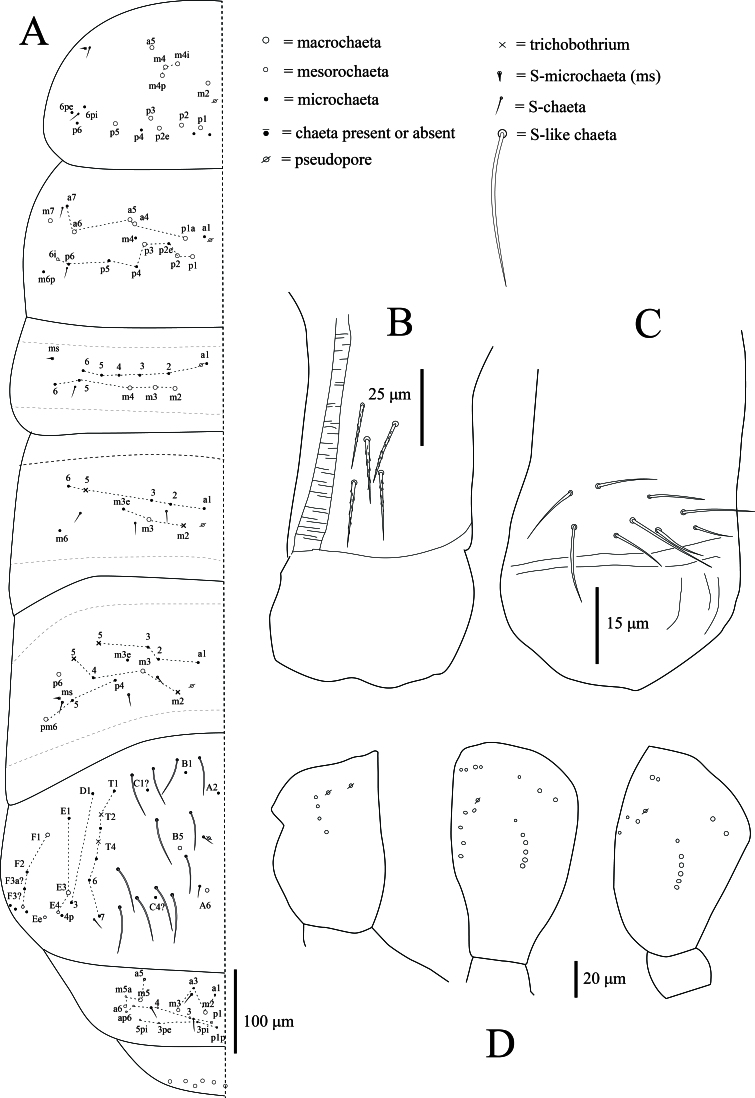
*Alloscopus ramanai* **sp. nov. A** Body chaetotaxy (left side); **B** Anterior ventral tube; **C** Lateral flap; **D** Outer chaetotaxy of coxae I–III (left side).

**Figure 5.**
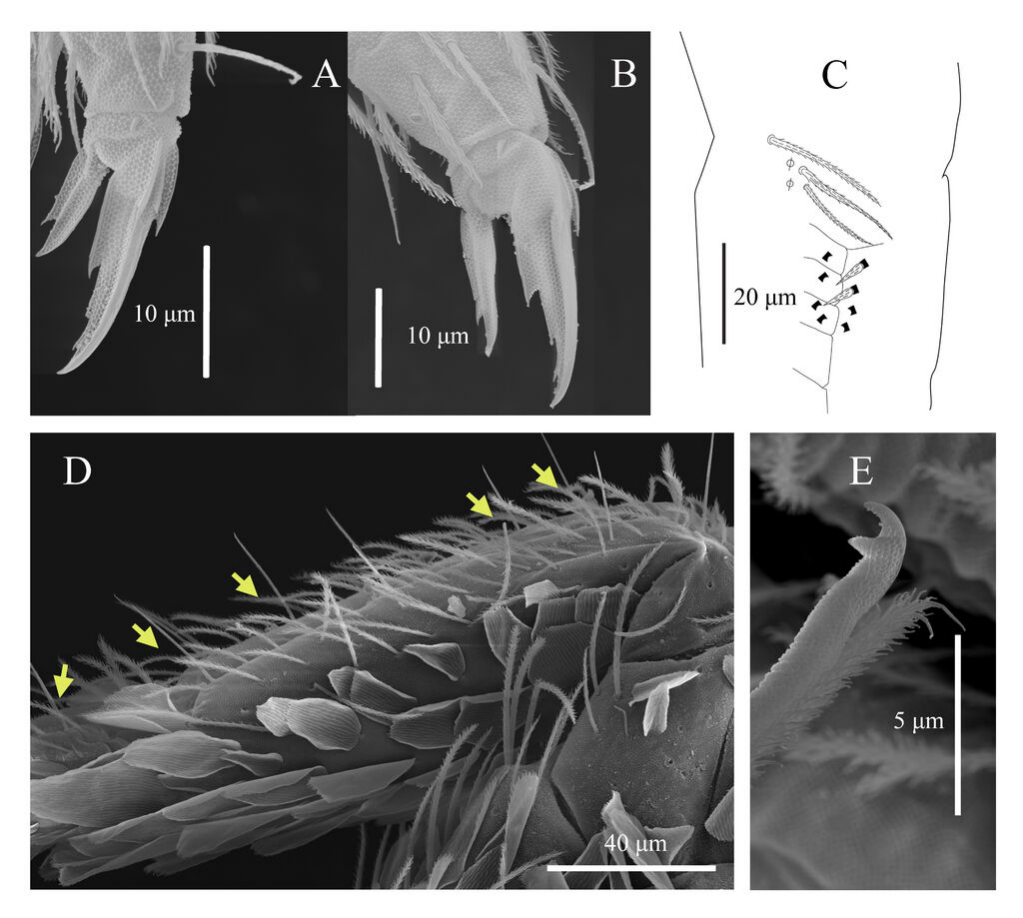
*Alloscopus ramanai* **sp. nov**. **A** Claw complex I (SEM); **B** Claw complex III (SEM); **C** Manubrial plaque and proximal part of dens and rows of spines; **D** SEM photograph of dorso-lateral manubrium and dens basally showing rows of smooth chaetae (yellow arrows); **E** SEM photograph of Mucro.

**Figure 6.**
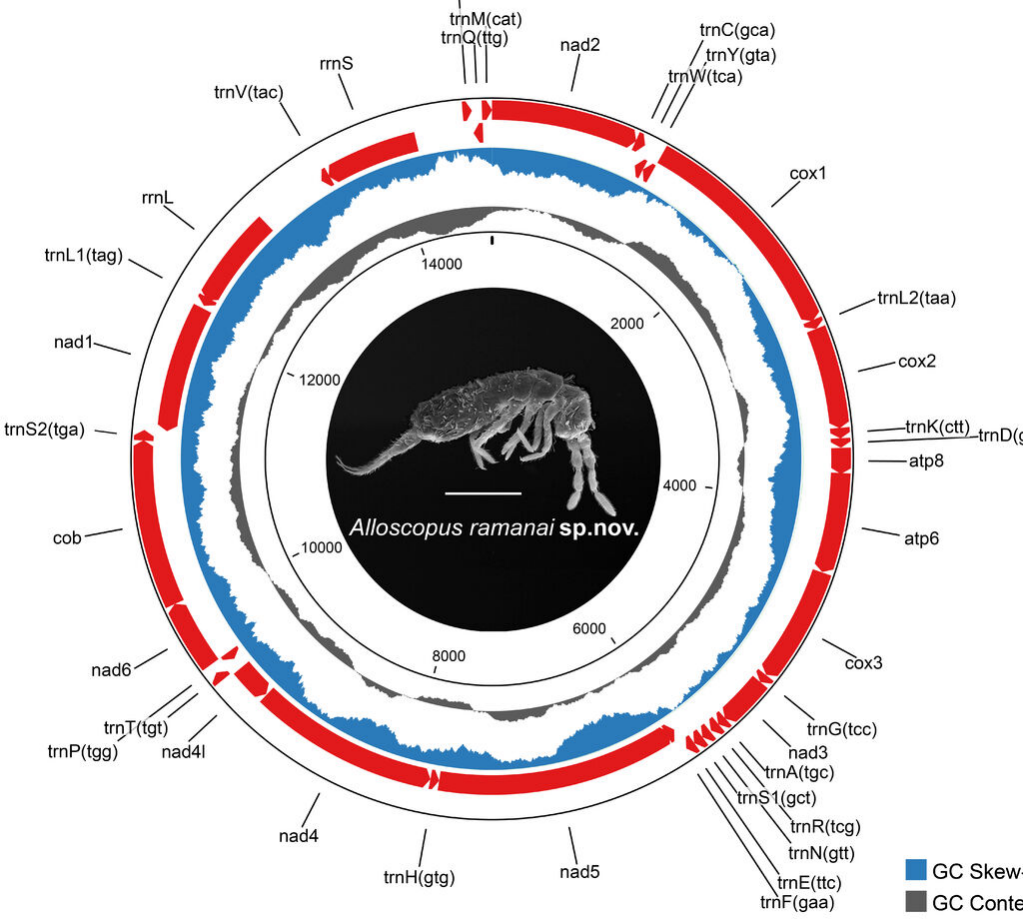
Mitochondrial genome of *Alloscopus ramanai* **sp. nov.** Genes are shown in red. The outermost and the next inner circles represent plus and minus strands, respectively. GC skew and GC content are shown in blue and grey, respectively. Scale bars = 0.5 mm.

Medium-size Orchesellidae, with body length (head + trunk) ranging from 1.1–1.4 mm. Scales present on both sides of Ant. I–II, on both sides of the head, on the entire body, on legs (coxa to femur) and on the ventral tube and furca. Body colour whitish with orange dots (Fig. 1A). Ocular patch present with small dark spot (Fig. 1A). Antennae shorter than body (Fig. 1A, Fig. 2A and Fig. 6). Eyes absent. Body slender, not bent nor humped at level of Th. II. Pseudopores present as round, flat discs, as large as mac sockets, present on various parts of body: antennae, head, tergites, coxae and manubrium. On antennae, psp located on tip of Ant. II and III (2 on each segment) (Fig. 2C). On head, 1+1 psp located laterally anterior to PAO (Fig. 3F). On tergites, 1+1 psp close to axis from Th. II to Abd. IV (Fig. 4A). On coxae, 1+1 psp on coxa I and II, located close to longitudinal rows of chaetae (Fig. 4D). On manubrium, 2+2 dorso-apical ones (Fig. 5C).

**Antennae**: Antennae elongated, with total length measuring 1.51–2.31 times cephalic diagonal and 0.34–0.48 times as long as body (head + trunk) (n = 4). Ant. I subdivided (Ia and Ib), Ant. III–IV annulated, except for their proximal and distal part (Fig. 1A, Fig. 2A and Fig. 6). Ant. II–III sometimes fused, not annulated. Ant. I(a+b): II: III: IV = 1:0.95–1.10: 1.26–1.36: 1.51–1.62 (Fig. 1A, Fig. 2A, Fig. 3H and Fig. 6). Antennal chaetae multiple, with ordinary chaetae, S-chaetae and scales (as described in Jantarit and Sangsiri (2020)and Jantarit et al. 2025. Scales oval to rounded and of medium size (11–13 x 16–24 μm). Smooth spiny mic present at base of antennae, with 4 dorsal and 3 ventral on Ant. Ia (Fig. 3H), 2 internal, 2 external and 1 ventral on Ant. II. Ant. II organ with 2 – 3 swollen S-chaetae distally (Fig. 2F). Chaetal type morphology of antennae not analysed in this work. Ant. III organ with typical 5 chaetae with two internal paddle-like chaetae in the middle (Fig. 2E). Subapical organite not distinctly knobbed, apical not enlarged, inserted dorsally near the tip of Ant. IV, with apical guard chaetae (Fig. 3C).

**Mouthparts**: Prelabral and labral chaetae 4/5, 5, 4, all smooth, acuminate, subequal, except chaetae of proximal row slightly longer than others. Four labral papillae, conical, minute (Fig. 2G and Fig. 3B). Ventral complex of labrum with two slightly asymmetrical multi-toothed combs and a pair of thin, sinuous, unequal tubules below. Maxillary outer lobe with 1 basal chaeta, simple maxillary palp, 4 sublobal appendages, all smooth (Fig. 2G and Fig. 3D). Labial palp with 5 smooth, acuminate proximal chaetae and 5 papillae (A = 0, B = 5, C = 0, D = 4, E = 4) (Fig. 2G and Fig. 3E). Labial papilla E with lateral process subcylindrical apically, not reaching apex of papilla (Fig. 3E). Mandible asymmetrical (right with 4 and left with 5 teeth) on all examined specimens.

**Ventral head chaetotaxy**: Chaetae of labial basis as M1m2rel1l2, chaetae M1 ciliated, m2, e and l2 smooth, subequal and longest, r and l1 smooth, subequal and shortest (Fig. 2G and Fig. 3A). Postlabial quadrangle (PLQ) with 4+4 weakly-serrated chaetae (Fig. 3A). Dense cover of scales and weakly-serrated chaetae.

**Dorsal head chaetotaxy**: Dorsal cephalic chaetotaxy with stable chaetae arrangement as in Fig. 3F. Head ‘An’ series with 8+8 chaetae, mac, ‘A’ series with 5+5, mac (A0, A2–A5), A1 as mic; ‘M’ series with 3+3 mac (M1–M3), sutural area with 7+7 mac (S0, S1, S3–6, S6i) and 2 unnamed mic between series ‘M’ and ‘S’; interocular series with 3+3 chaetae (p as mac, t and r as mic); postsutural area with 3+3 mic (Ps2–3 and 5); post-occipital anterior area with 1+1 mac (Pa5), 1+1 short cephalic tric (Pa6) and 1+1 unnamed mic laterally; post-occipital posterior area with 3+3 mic (Pp3 and Pp5–6); head laterally with several unnamed mac. Eyes absent. PAO shape irregular (three overlapping ovals) located just below antennal mac (Fig. 3F).

**Dorsal chaetotaxy**: Formulas for Th.II–Abd.V: psp formula as 1,1/ 1,1,1,1,0; tric formula as 0,0/0,2,3,2,0; ms formula as 1,0/1,0,1,0,0; sens formula as 2,2/1,3,3,3,3; mac formula as 10,8/3,1,3,4,3. Mac arrangement stable; multiplets anteriorly on Th.II. Th.II with 5+5 anterior central mac (a5, m2, m4, m4i, m4p) and 5+5 posterior mac (p1–3, p5, p2e). Th.III with 6+6 central mac (a2, a4–5, p1–3) and 2+2 lateral mac (a6, m7). Abd.I with 3+3 central mac (m2–4). Abd.II with 1+1 central mac (m3). Abd.III with 1+1 central mac (m3) and 2+2 lateral mac (p6, pm6). Abd.IV with 2+2 central mac (A3, A6); 2+2 lateral mac (E3, F1); and at least 12+12 S-like chaetae. Abd. V with 2+2 central mac (m2–3) and 1+1 lateral mac (m5) (Fig. 4A).

**Legs**: with ordinary ciliated (mes to mac), smooth chaetae and scales; mic not seen. Scales cover the coxa, trochanter and femur. Tita of leg III is slightly longer than tita of legs I and II. Subcoxa of leg I with 4+4 mac, subcoxa of leg II with 4–5+4–5 mac and 4+4 mes, subcoxa of leg III with 3–4+3–4 mes anteriorly and 3–4+3–4 mac posteriorly. Coxa of leg I with 2 proximal psp, 4–5 anterior mes and 4 posterior mac; coxa of leg II with 5 mac in anterior row, 4–5 mac in posterior row and 1 proximal psp in between, at least row of 2–3 mes posteriorly; coxa of leg III with at least 7 chaetae: 1 mac, 2 mes anteriorly, (5 mac, 1–2 mes posteriorly) and 1 proximal psp (Fig. 4D). Trochanteral organ with 17–18 smooth, straight, unequal spine-like chaetae (n = 3) (Fig. 3J). Distal whorl of tita with 9–10 subequal ciliated chaetae, irregularly arranged and a thin, acuminate, clavate tenent hair. Tita with a long, smooth chaeta latero-distally. Presence of one smooth chaeta on tita of leg III latero-distally (one specimen with two smooth chaetae). Ventro-distal smooth chaeta of tita III thin, erected, pointed, longer than tenent hair or unguiculus (Fig. 5B). Pretarsal minute on the anterior and posterior sides. Unguis with one strong outer tooth and one lateral tooth on each side, inner edge with paired basal teeth and two unpaired teeth. Unguiculus ~ 0.5x as long as the inner edge of unguis, slightly swollen baso-internally, pointed apically with a large outer tooth (Fig. 5A–B).

**Ventral tube**: Ventral tube about 2 times longer than wide, with scales on the posterior side. Anteriorly with 5+5 subequal ciliated chaetae (Fig. 2D and Fig. 4B). The posterior ventral tube not clearly seen in all specimens. Lateral flaps with 11+11 smooth chaetae (Fig. 4C).

**Furcal complex**: Tenaculum with 1 smooth chaeta and 4 large teeth of decreasing size from the basal to distal one on each ramus (Fig. 3I). Ratio of manubrium: dens: mucro = 5.11:9:1 (n = 5). Mucrodens is about 2.0 times longer than the manubrium (n = 5). Manubrium dorsally densely covered with ciliated mes with a row of 5–6+5–6 smooth chaetae on each side (Fig. 5D). Manubrial plate with 2+2 psp and 3 ciliated chaetae (Fig. 5C). Manubrium ventrally with densely medium-sized scales. Dens curved, tapering, constituted of a rather short basal part hardly crenulated, long medial part with well-defined dorsal crenulations and short, thinner, smooth distal part, smooth section about 4 times as long as the mucro. Dens basally with 1+1 row of 4–5 finely ciliated spines on the inner side (normally with 5+5 with sometimes asymmetry) (Fig. 5C). Mucro bidentate, without basal spine (Fig. 5E).

**Genital plate**: Female with 2+2 smooth mic, 1 pair on anterior and posterior lobes (Fig. 3G). Male was not found in this study.

#### Diagnosis

*Alloscopus ramanai* **sp. nov.** exhibits the morphological similarity to *A. tetracanthus* Börner 1906, a species reported from Australia, Indonesia, Malaysia, Singapore, Papua New Guinea, India, New Britain, Micronesia and Thailand (Chiang Mai Province). Both species share several morphological characters, including a dark red eye patch with reddish to dark dot pigmentation, a semi-divided PAO, labial basis M1(m)m2rel1l2, four spiniform labral papillae, 4–5 central and 4–5 posterior mac on Th. II, six central mac on Th. III, two central mac on Abd. IV, 0–2 inner unpaired ungual teeth, the presence of teeth on the unguiculus, smooth chaetae on the tibiotarsi, a similar number of chaetae on the manubrium and 4–7 spines on the dens. However, *Alloscopus ramanai* **sp. nov.** can be distinguished from *A. tetracanthus* by the absence of eyes (vs. 1+1), orange dot pigmentation (vs. lack of pigmentation) and the presence of five macrochaetae on the “A” series of the dorsal head chaetotaxy (vs. four). Furthermore, *A. ramanai* **sp. nov.** can be separated from all other Thai *Alloscopus* species by the following unique combination of characters: (1) two lateral mac on Abd. III (vs. one in other species); (2) two lateral mac on Abd. IV (vs. four in other species); and (3) five chaetae on the anterior face of the ventral tube (vs. 8+8 in *A. tetracanthus*, 7–9+7–9 in *A. whitteni*, 9+9 in *A. namtip*, 6+6 in *A. jantapasoae* and 7+7 in *A. sago*). Diagnostic characters of this new species and all known six species in Thailand are provided in Table 1.

#### Etymology

The new species was collected in the vicinity of the Rama 9 Museum, part of the National Science Museum, Thailand, which serves as the locality. The specific epithet ramanai is derived from the name of the Museum and is used as a noun in apposition, honouring the institution.

#### Distribution

Pathum Thani, Thailand (central part).

#### Ecology

The new species was found and collected from the leaf litter and soil near a lotus pond (Bueng Bua) at a forest plantation, located in a protected zone managed by the staff of the National History Museum Thailand (NSM) at the Rama 9 (Ramanai) building in Pathum Thani Province. The plantation is designated for the education and conservation of tropical plant species. The area supports a heterogeneous assemblage of vegetation, including wetland and mangrove-associated taxa (e.g. *Rhizophora* spp., *Sonneratia* sp.) as well as terrestrial tropical plant families such as Anacardiaceae and Fabaceae (Fig. 1B–C). According to Good’s biogeographic classification (Good 1974), central Thailand lies within the Indo-Malayan (Oriental) Region of the Palaeotropical Kingdom. The local climate is classified as tropical savannah with a dry winter (Aw) under the Köppen-Geiger climate classification system (Kottek et al. 2006).

## Identification Keys

### Identification keys of *Alloscopus* in Thailand

**Table:** 

1	Labrum with two spiniform labral papillae	2
–	Labrum with four spiniform labral papillae	3
2	Ant. IV with apical pin chaeta; PAO with oval shape; labial basis: M1m2el1l2; Th.II with 6+6 posterior mac	*A. thailandensis* Mari- Mutt, 1985
–	Ant. IV without apical pin chaeta; PAO with semi-divided shape; labial basis: M1m2rEl1(l2); Th.II with 5+5 posterior mac	*A. namtip* Jantarit & Sangsiri, 2020
3	Pigmentation present (orange dots)	4
–	Pigmentation absent (white)	6
4	Head “A” series with 5+5 (A0, A2–A5); Th.II with 11+11 mac; Abd.IV with 7+7 mac; absence of smooth chaetae on tibiotarsi	*A. jantapasoae* Jantarit, Nilsai & Manee, 2025
–	Head “A” series with 4+4 (A0, A2–A4); Th.II with 10+10 mac	5
5	Th.III present 2+2 lateral mac; Abd.IV with 2+2 lateral mac (E3, F1); anterior face of ventral tube with 5+5 chaetae; dorsal part of manubrium with 3+3 ciliated chaetae	*A. ramanai* Nilsai, Jantarit & Jaitrong **sp.nov.**
–	Th.III present 1+1 lateral mac; Abd.IV with 4+4 lateral mac (E1, E3–4, Ee); anterior face of ventral tube with 7–9+7–9 chaetae; dorsal part of manubrium with 4+4 ciliated chaetae	*A. whitteni* Jantarit & Sangsiri, 2020
6	Eyes absent; Head “A” series with 5+5 (A0, A2–A5); trochanteral organ with 18–21 smooth chaetae	*A. sago* Jantarit & Manee, 2025
–	Eyes 1+1; Head “A” series with 4+4 (A0, A2–A4); trochanteral organ with 15 smooth chaetae	*A. tetracanthus* Börner, 1906

## Analysis

### Characterisation of the complete mitogenome and position of Alloscopus ramanai sp. nov.

The complete mitochondrial genome of *Alloscopus ramanai* **sp. nov.** is 14,757 bp in length and contains the typical set of 37 genes (Table in Suppl. material 1), including 13 PCGs, 22 transfer RNA (tRNA) genes and two ribosomal RNA (rRNA) genes (Fig. 6, Table 2). The 13 PCGs range in length from 168 bp (*atp8*) to 1,732 bp (*nad5*). Several genes exhibit overlaps or are separated by short intergenic spacer regions. The majority of genes are encoded on the positive strand of the mitogenome, except for *nad5*, *nad4*, *nad4l* and *nad1*, which are located on the negative strand. The overall nucleotide composition is 37.38% A, 33.11% T, 11.61% G and 17.90% C.

**Figure 7.**
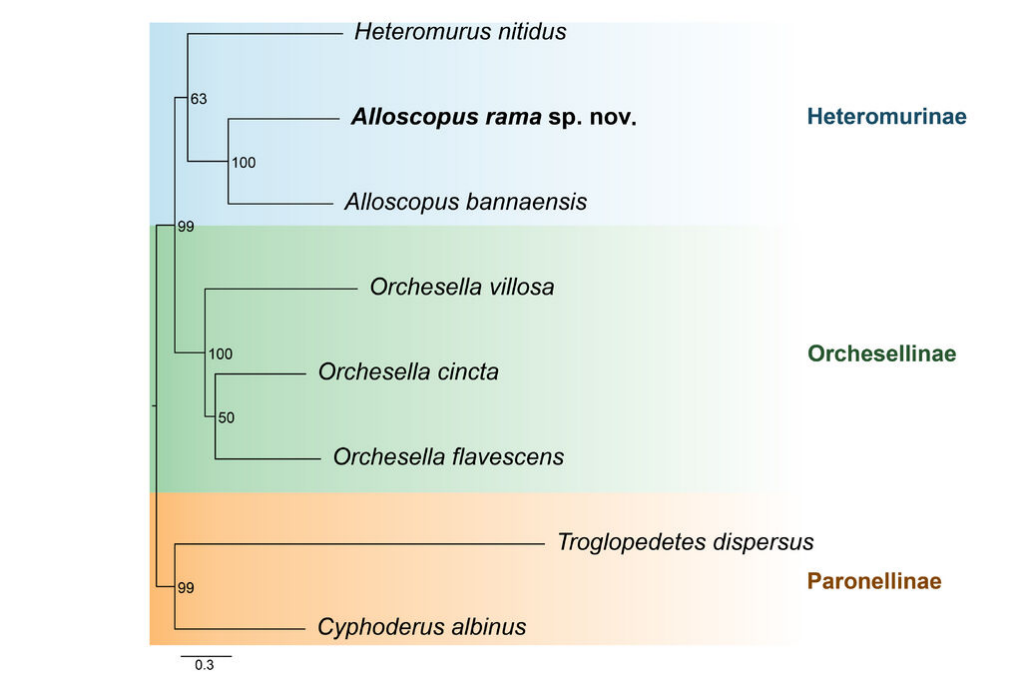
Maximum Likelihood phylogenetic tree of *Alloscopus ramanai* **sp. nov.** based on 13 protein-coding genes in mitochondria. The number at each node represents the bootstrap value.

Phylogenetic analysis, based on 13 mitochondrial protein-coding genes, using a Maximum Likelihood approach, places *A. ramanai* **sp. nov.** within the subfamily Heteromurinae, forming a sister relationship with *A. bannaensis*. This clade is further grouped with *Heteromurus nitidus* (Fig. 7). The analysis also clearly shows separation amongst the three subfamilies Heteromurinae, Orchesellinae and Paronellinae, each forming a well-supported monophyletic group with high bootstrap values.

**Figure 8.**
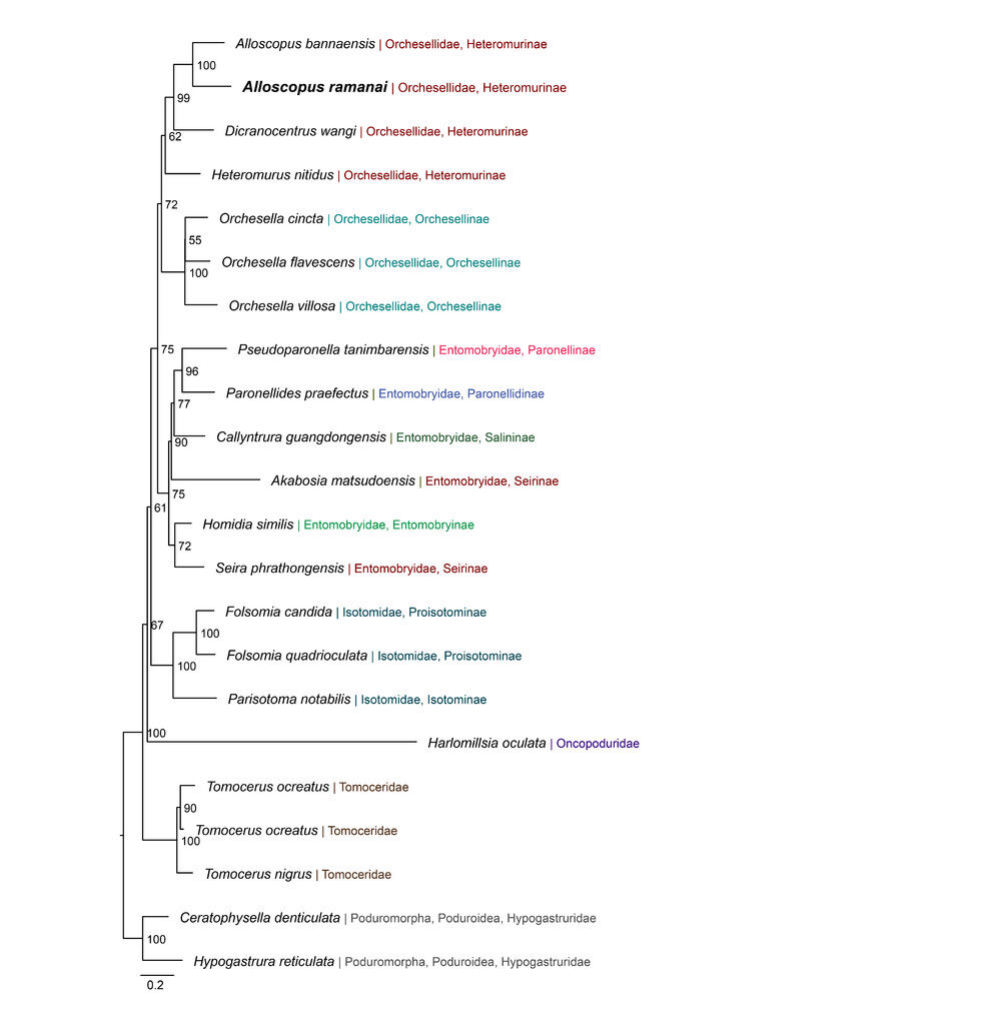
Maximum-Likelihood phylogeny of Entomobryomorpha inferred from a concatenated dataset of mitochondrial COI and 16S rRNA and nuclear 28S rRNA sequences (total length 1,822 bp). Node support values indicate ultrafast bootstrap percentages (1,000 replicates). *Ceratophysella denticulata* and *Hypogastrura reticulata* (Poduromorpha) were used as outgroups. *Alloscopus ramanai* **sp. nov.** is recovered within *Alloscopus* as sister to *A. bannaensis* (ultrafast bootstrap, 100).

Maximum-Likelihood inference from concatenated COI, 16S rRNA and 28S rRNA dataset recovered *A. ramanai* **sp. nov.** within the genus *Alloscopus*, forming a strongly-supported sister relationship with *A. bannaensis* (ultrafast bootstrap, 100; Fig. 8). This *Alloscopus* clade was placed within Heteromurinae and grouped with other Heteromurine taxa included in the analysis, whereas deeper relationships amongst major lineages showed variable bootstrap support. This dataset, with expanded taxon sampling, places *A. ramanai* **sp. nov.** within *Alloscopus* and recovers a strongly-supported sister relationship with *A. bannaensis*. Overall, the expanded multi-locus dataset supports the placement of *A. ramanai* **sp. nov.** in *Alloscopus*, while indicating that denser sampling will be required for robust tests of subfamily-level monophyly and inter-subfamily relationships.

## Discussion

**Taxonomic note and phylogenomic position of *Alloscopus ramanai* Nilsai, Jantarit & Jaitrong, sp. nov.**

This study provides new insights into the taxonomy of *Alloscopus* within the tribe Heteromurini by integrating morphological and molecular evidence. The discovery of *Alloscopus ramanai* Nilsai, Jantarit & Jaitrong, **sp. nov.** in central Thailand suggests that the genus has a broader distribution within the country than previously recognised. However, because the species is currently known from a limited sampling area, its distribution cannot yet be regarded as restricted to a single locality.

Morphological examination of the available specimens of *A. ramanai* **sp. nov.** revealed only limited intraspecific variation. Minor differences were observed in body length (1.1–1.4 mm), antenna-to-head length ratio (1.5–2.3 times), the number of spines on the dens (4–5) and number of smooth chaetae on the trochanteral organ (17–18). In contrast, key diagnostic characters such as dorsal chaetotaxy pattern of central area of Th. II–Abd. IV, the number of inner unpaired teeth on the unguis (2) and the labial chaetae (M1m2rel1l2), were consistent across all examined specimens. Several stable morphological characteristics further indicate low variability within the genus *Alloscopus*. Notably, a highly conserved chaetotaxic pattern is observed, with 3+3 mac (m2–4) on Abd. I and 1+1 central mac (m3) on Abd. II–III. This configuration is consistent across all valid species of *Alloscopus*, including *A. ramanai* **sp. nov.** and represents a set of constant diagnostic characters for the genus (Cipola et al. 2016, Zhang et al. 2019). In contrast, variation in chaetotaxy on the lateral Abd. III–IV appears to be informative for species-level identification. *Alloscopus ramanai* **sp. nov.** shows a stable chaetotaxic pattern on the lateral Abd. III–IV compared with other species; however, comparable data for the closely-related species, *A. tetracanthus*, are currently unavailable. Additional comparative data are, therefore, required to support the distinction between these two species more robustly.

The taxonomic status of *A. tetracanthus* remains questionable. Previous studies have reported notable variation amongst populations, including differences in eye presence across localities, suggesting substantial morphological heterogeneity. Such variation raises the possibility that records attributed to *A. tetracanthus* may represent more than one species. Given that *A. tetracanthus* has been reported from a wide geographic range, based on specimens from diverse sources (Börner 1906, Mari-Mutt 1982, Cipola et al. 2016), a comprehensive taxonomic revision, based on material from the type locality, is strongly recommended to clarify its species boundaries and confirm its taxonomic validity.

It should be noted that the taxonomic significance of the post-antennal organ (PAO), which has long been regarded as a diagnostic character of *Alloscopus* (Cipola et al. 2016, Zhang et al. 2019, Alviola et al. 2024, Jantarit et al. 2025), warrants re-evaluation across species of the genus. This structure is present in all currently valid *Alloscopus* species, including *A. ramanai* **sp. nov.**, suggesting that its presence alone may be insufficient for species-level discrimination. Although the PAO has been studied in detail in several Collembola groups, such as Symphypleona (Dallai 1971), Onychiuridae (Pomorski and Skarzynski 1992, Weiner 1996) and Isotomidae (Bellinger et al. 1996-2025, D'Haese 2003), its functional role remains poorly understood. Within *Alloscopus*, detailed comparative information on PAO morphology is still limited. Future studies should therefore emphasise intraspecific and interspecific variation in this character, including the number, arrangement and precise shape of PAO elements, as such features may provide additional informative characters for species identification.

The availability of a complete mitochondrial genome for *Alloscopus ramanai* **sp. nov.** is important because mitogenomes provide a well-annotated, comparable genomic unit for cross-species studies of genome architecture, nucleotide composition bias, codon usage and evolutionary rate heterogeneity in Collembola. As a reference sequence, it also supports marker development and primer design for mitochondrial loci used in taxonomy and phylogeography. In addition, it strengthens DNA barcoding frameworks for accurate identification of closely-related and potentially cryptic species, consistent with prior Collembola mitogenome reports that emphasise their value for identification and phylogenetic study. Mitogenomic datasets have also been explicitly evaluated for reconstructing Collembola phylogeny, supporting their continued utility as genomic resources as taxon sampling expands.

The gene arrangement of *A. ramanai* **sp. nov.** is similar to previously reported mitogenomes of Collembola, such as *Lepidocyrtinus boneti* (Godeiro et al. 2023a), *Seira brasiliana*, *S. dowlingi*, *S. phrathongensis* (Bellini et al. 2024) and *Troglopedetes meridionalis* (Nilsai et al. 2025), supporting a largely conserved mitogenome organisation within the group. This is expected because mitochondrial gene re-arrangements in springtails are often lineage-specific and tend to be most informative at lower taxonomic levels, while Entomobryomorpha, in particular, shows strong conservation of the ancestral Pancrustacea gene order across most available genomes (Cucini et al. 2020). Earlier comparative work likewise recognised the ancestral Pancrustacea gene order as the most frequently observed arrangement in Collembola (Carapelli et al. 2014).

Phylogenetic analysis, based on 13 mitochondrial protein-coding genes, using a Maximum-Likelihood approach, places *A. ramanai* **sp. nov.** within Heteromurinae, as sister to *A. bannaensis*. However, because complete mitogenomes are only available for a subset of described springtail diversity, relationships inferred from mitochondrial-only sampling should be interpreted cautiously and they may be refined as additional taxa become available (Carapelli et al. 2014).

In terms of nucleotide composition, *A. ramanai* **sp. nov.** shows an A+T bias (70.49%), which falls within the reported range for Collembola mitogenomes (60.4% to 74.8%) and is comparable to other entomobryid mitogenomes, such as *Homidia koreana* (68.4% A+T) (Cucini et al. 2020, Lee et al. 2021). Across earlier complete collembolan mitogenomes, A+T content varies substantially, for example, 75% in *Folsomota octooculata* (reported as the highest amongst ten genomes at that time) and 69% in *Sminthurus viridis* (reported as the lowest), indicating that the A+T richness observed in *A. ramanai* **sp. nov.** is typical rather than exceptional (Carapelli et al. 2014). Such compositional heterogeneity is often linked to asynchronous and asymmetric replication of mitochondrial DNA, during which one strand persists longer as single-stranded DNA and may experience stronger deamination-driven mutational biases. It can also interact with codon-position effects, including T enrichment at second codon positions in mitochondrial protein-coding genes (Carapelli et al. 2014, Leo et al. 2019).

## Supplementary Material

XML Treatment for Alloscopus ramanai

E7DD6773-02F1-5AAE-8F74-1E32AF45B766 10.3897/BDJ.14.e173157.suppl1 Supplementary material 1 Mitochondrial genome features Data type mitochondrial genome features Brief description The length of each gene arrangement in the mitochondrial genome of *Alloscopus ramanai* sp. nov. File: oo_1504895.xlsx https://binary.pensoft.net/file/1504895 Matsapume Detcharoen, Areeruk Nilsai, Sopark Jantarit, Weeyawat Jaitrong, Tadsanai Jeenthong

50B9B982-04BC-58E1-9533-789A60FD51EF 10.3897/BDJ.14.e173157.suppl2 Supplementary material 2 GenBank accession numbers Data type GenBank accession codes Brief description GenBank accession numbers of COI, 16S rRNA and 28S rRNA sequences for all Collembola taxa included in the Maximum-Likelihood phylogeny of this study. File: oo_1504896.xlsx https://binary.pensoft.net/file/1504896 Matsapume Detcharoen, Areeruk Nilsai, Sopark Jantarit, Weeyawat Jaitrong, Tadsanai Jeenthong

## Figures and Tables

**Table 1.** Morphological comparisons amongst Thai *Alloscopus* species. The symbol ‘?’ indicates missing or uncertain information in the sources. Characters shown in bold represent traits unique to the new species.

**Characteristics/species**	***A. tetracanthus***	***A. thailandensis***	***A. whitteni***	***A. namtip***	***A. jantapasoae***	***A. sago***	***A. ramanai* sp.nov.**
Body length (mm)	1.6	1.7	1.7	1.6	1.3–1.7	1.2–1.5	1.1–1.4
Ant./head (mm)	2.2	?	1.5	2	1.59–2.0	1.8–2.5	1.5–2.3
Eye patch	absent to reddish-brown	dark red	dark red	dark red	dark red	dark red	dark
Number of eyes	1+1	0+0	?	?	0+0	0+0	0+0
Pigmentation	absent	?	orange dots	absent	orange dots	absent	orange dots
Ant. IV apical pin chaeta	present or absent	present	absent	absent	absent	absent	absent
PAO shape	semi-divided	oval	semi-divided	semi-divided	semi-divided	semidivided	semidivided
Head Chaetotaxy							
An	?	3?	8	9	9	8	8
A	4 (A0, A2–A4)	5 (A0, A2–A5)	5 (A0, A2–A5)	4 (A0, A2–A4)	4 (A0, A2–A4)	5 (A0, A2–A5)	5 (A0, A2–A5)
Labial basis	M1(m1)m2rel1l2	M1m2?el1l2	M1m2rel1(l2)	M1m2rEl1(l2)	M1m2_el1l2	M1m2rel1l2	M1m2rel1l2
Chaetae of PLQ	?	smooth	weakly serrated	weakly serrated	weakly serrated	weakly serrated	weakly serrated
Length of the l.p. to papilla E	1/3	1/2	1/2	1/3	3/4	3/4	1/2
Appendices in papilla E	?	3	5	5	5	5	5
Spiniform labral papillae	4	2	4	2	4	4	4
Chaetotaxy of Th. II	9–10+9–10	12+12	10+10	11+11	11+11	9+9	10+10
central mac	4–5+4–5 (a5, m2, m4, (m4p), m4i)	6+6 (a5, m2, m2i, m4, m4p, m4i)	5+5 (a5, m2, m4, m4p, m4i)	6+6 (a5, m2, m2i, m4, m4p, m4i)	6+6 (a5, m2, m2i, m4, m4p, m4i)	4+4 (a5, m2, m4, m4p)	5+5 (a5, m2, m4, m4p, m4i)
posterior mac	4–5+4–5 (p1–2, (p3), p4–5)	6+6 (p1–2, p4–6, 1p)	5+5 (p1–3, p2e, p5)	5+5 (p1–3, p2e, p5)	5+5 (p1–3, p2e, p5)	5+5 (p1–3, p2e, p5)	5+5 (p1–3, p2e, p5)
Chaetotaxy of Th. III	6+6?	7+7	7+7	7+7	7+7	7+7	8+8
central mac	6+6 (p1–3, p1a, a4–5)	6+6 (p1–3, p1a, a4–5)	6+6 (p1–3, p1a,, a4–5)	6+6 (p1–3, p1a, a4-5)	6+6 (p1–3, p1a, a4–5)	6+6 (p1–3, p1a, a4–5)	6+6 (p1–3, a2, a4–5)
lateral mac	?	1+1 (a6)	1+1 (a6)	1+1 (a6)	1+1 (a6)	1+1 (a6)	2+2 (a6, m7)
Chaetotaxy of Abd IV	?	?	6+6	6+6	7+7	6+6	4+4
central mac	2+2 (A6, B5)	2+2 (?)	2+2 (A6, B5)	2+2 (A6, B5)	3+3 (A6, B6, C2)	2+2 (A6, B5)	2+2 (A6, B5)
lateral mac	?	?	4+4 (E1, E3–4, Ee)	4+4 (E1, E3–4, Ee)	4+4 (E1, E3–4, Ee)	4+4 (E1, E3–4, Ee)	2+2 (E3, F1)
Ungual inner unpaired teeth	0–2	1–2	0	2	0–1 (tiny)	0	2
Teeth in unguiculus	present	present	present	present	present	present	present
Smooth chaetae on tibiotarsi	present	absent	present	absent	absent	present	present
Smooth chaetae on trochanteral organ	15	15	12–20	25–32	16–22	18–21	17–18
Chaetae on ventral tube							
anterior face	8+8	?	7–9+7–9	9+9	6+6	7+7	5+5
posterior face	16	?	12	23	10–11+10–11	7–8+7–8	?
lateral flap	?	?	11–12+11–12	12+12	11+11	11+11	11+11
Chaetae on manubrium	3+3	4+4	4+4	4+4	3+3	5+5	3+3
Number of spines on dens	4–7	3–6	4–6	4–6	3–5	4–5	4–5
Ecology	leaf litter, forests, tea field	leaf litter, soil, roots, tree bark	cave	cave	cave	inside the bark of Sago palm trees	leaf litter, humus
Distribution	Chiang mai	Chiang mai	Phangnga	Suratthani	Trang	Phathalung	Pathum Thani
Sources (1906–2025)	Börner (1906), Mari-Mutt (1977) Prabhoo 1971 Yoshii and Suhardjono 1989 Cipola et al. 2016, Jantarit et al. 2016	Mari-Mutt 1985, Cipola et al. 2016	Jantarit and Sangsiri 2020		Jantarit et al. 2025		This study

**Table 2.** Thirteen protein-coding genes identified in *Alloscopus ramanai* **sp. nov.** mitochondrial genome.

**Symbol**	**Strand**	**Protein**
*nad2*	+	NADH dehydrogenase subunit 2
*cox1*	+	Cytochrome c oxidase subunit 1
*cox2*	+	Cytochrome c oxidase subunit 2
*atp8*	+	ATP synthase F0 subunit 8
*atp6*	+	ATP synthase F0 subunit 6
*cox3*	+	Cytochrome c oxidase subunit 3
*nad3*	+	NADH dehydrogenase subunit 3
*nad5*	-	NADH dehydrogenase subunit 5
*nad4*	-	NADH dehydrogenase subunit 4
*nad4l*	-	NADH dehydrogenase subunit 4L
*nad6*	+	NADH dehydrogenase subunit 6
*cob*	+	Cytochrome b
*nad1*	-	NADH dehydrogenase subunit 1
